# Immobilisation in a collar and cuff with high elbow flexion is a safe and effective treatment option to reduce and immobilise Gartland type II supracondylar fractures

**DOI:** 10.1308/rcsann.2024.0071

**Published:** 2024-09-03

**Authors:** RY Yap, L Bommireddy, A Firth, BA Marson, K Price, D Lawniczak

**Affiliations:** Nottingham University Hospitals NHS Trust, UK

**Keywords:** Elbow fracture, Supracondylar, Distal humerus fracture

## Abstract

**Introduction:**

This study aimed to report the proportion of children requiring subsequent surgical intervention, rate of complications and radiologic outcomes following collar and cuff immobilisation with high elbow flexion (>90°) for Gartland type II supracondylar fractures.

**Methods:**

A retrospective case series of consecutive patients aged <18 years with Gartland type II fractures treated at a level 1 trauma centre from December 2020 to April 2023 was conducted. The need for surgical intervention and complications were recorded from electronic clinical notes. The initial, post-immobilisation and final Baumann's angle and lateral humeral–capitellar angle (LHCA) were measured and compared.

**Results:**

In total, 42 patients were included in this study. Thirty-four were treated definitively in a collar and cuff with a mean elbow flexion of 109.4°. Two patients underwent closed reduction and Kirschner wire fixation. No patient required subsequent corrective osteotomy. There were no cases of recorded complications. Immobilisation in a collar and cuff with >90° elbow flexion was associated with a significant improvement in the mean LHCA (initial 80.9° vs final 65.6°, *p* < 0.001). There was no significant change in the LHCA post immobilisation in a collar and cuff until final radiographic follow-up (post immobilisation 68.3° vs final 65.6°, *p*=0.274).

**Conclusions:**

Immobilisation in a collar and cuff with high elbow flexion is a safe and effective nonoperative treatment method to reduce and immobilise Gartland type II supracondylar fractures. Surgical treatment could be reserved for cases with unsatisfactory alignment or early loss of reduction following attempted nonoperative treatment.

## Introduction

Supracondylar fractures of the humerus account for around 55–80% of all paediatric elbow fractures, with an estimated annual incidence of 177.3 per 100,000 children.^1^ These injuries are commonly categorised according to the Gartland's classification system.^2^ Gartland type I fractures are undisplaced fractures, in which the anterior humeral line (AHL) intersects the capitellum.^2^ Gartland type II fractures are moderately displaced fractures, with the AHL passing anterior to the capitellum but with an intact posterior cortex.^2^ Type II fractures can be subcategorised into IIA and IIB based on the absence and presence of malrotation, respectively.^3^ Gartland type III fractures are completely displaced with no cortical contact.^2^

Although surgeons generally agree that type I fractures could be managed nonoperatively and type III fractures require surgical fixation, there is a lack of consensus on the indications and methods for nonoperative and operative treatment for type II fractures.^1,4^ Operative treatment for type II fractures has gained popularity in recent years. According to an epidemiological study conducted in Finland, the proportion of type II fractures that were managed operatively increased from 5.9% to 37.1% from the year 2000 to 2009.^5^ O'Hara *et al* proposed that although type IIA fractures could be managed nonoperatively, type IIB fractures are more unstable and require Kirschner wire fixation.^6^ Some authors have recommended that all displaced type II fractures should be fixed to avoid the risk of neurovascular compromise and loss of alignment that could be associated with nonoperative treatment in a cast.^7,8^

Several studies have reported satisfactory outcomes with nonoperative treatment for Gartland type II fractures.^9–13^ Hadlow *et al* found that fixation of all type II fractures would result in an unnecessary surgical procedure in 77% of patients.^12^ Similarly, Parikh *et al* reported that 72% of patients maintained alignment following closed reduction and cast immobilisation.^13^ They recommended that surgical fixation should be reserved for cases that lose reduction after an attempt at closed reduction and casting.^13^ The potential benefits of surgical intervention need to be balanced against its risks, such as iatrogenic neurovascular injury, pin-site infection and compartment syndrome.^1^

At our institution, the first-line treatment for all closed Gartland type II supracondylar fractures without neurovascular compromise is by immobilisation in a collar and cuff with an elbow flexion of >90°. This study aims to report the proportion of children requiring subsequent surgical intervention, rate of complications, and radiologic outcomes associated with this treatment method.

## Methods

We conducted a retrospective case series of consecutive paediatric Gartland type II supracondylar fractures treated at a level 1 trauma centre between December 2020 and April 2023. All children aged under 18 years with a Gartland type II supracondylar fracture were eligible for inclusion. Exclusion criteria included open fractures, neurovascular compromise at presentation, concomitant ipsilateral upper limb injuries, lack of adequate radiographs after treatment and those initially treated or followed up at a different centre.

This study was approved by the local audit office (registration number: 21-420C) and data were handled according to the General Data Protection Regulation guidelines. Patients were identified from a local trauma registry. Data were collected from electronic clinical notes and the Picture Archiving and Communication System (PACS) using a standardised proforma. Information on sex, age, laterality of injury, mechanism of injury, treatment method, complications, date of latest radiographic and clinic follow-up was recorded. Mechanism of injury was categorised into high energy (road traffic accidents, fall from height) and low energy (sports and recreational, fall from standing height). Gartland's classification was assessed independently by two researchers (RY and LB). Conflicting Gartland's classifications were resolved with a senior researcher (AF) review. We did not subclassify type II fractures into IIA and IIB because of the low interobserver reliability demonstrated with the Wilkins-modified Gartland's classification system.^14^

**Figure 1:**
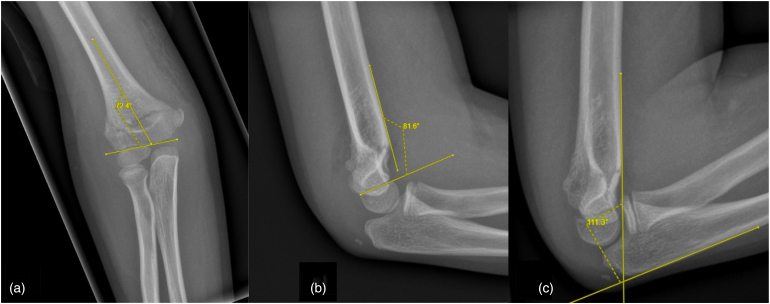
Methods for measuring radiologic parameters: (a) Baumann's angle, (b) lateral humeral–capitellar angle, (c) humeral–ulnar angle.

Radiologic parameters were measured on PACS (Figure 1). Baumann's angle was measured as the angle between the long axis of the humeral shaft and the capitellum physis on anteroposterior (AP) radiographs.^15^ The normal Baumann's angle is 71.5 ± 6°, with a minimal clinically important difference (MCID) of 6°.^15,16^ The lateral humeral–capitellar angle (LHCA) was measured as the angle between the anterior border of the distal humeral shaft and the capitellar physis on lateral radiographs.^15^ The normal LHCA is 50.8 ± 6.2°, with a MCID of 10°.^15,16^ The degree of elbow flexion, represented by the humeral–ulnar angle, was measured as the angle between the line along the anterior border of the distal humeral shaft and the line along the posterior border of the ulna on lateral radiographs.

Patients were managed in accordance with the British Orthopaedic Association Standards for Trauma and Orthopaedics guidelines for supracondylar fractures.^17^ The first-line treatment for closed Gartland type II supracondylar fractures with no neurovascular compromise at our institution was immobilisation in a collar and cuff with >90° of elbow flexion for a total of 3 weeks. This was achieved by moving the hand of the injured limb towards the contralateral shoulder. Patients were instructed to wear the collar and cuff under clothes. This facilitated additional protection by keeping the injured arm close to their trunk and minimised the risk of inadvertent manipulation of the fracture when dressing. Initial analgesic cover for collar and cuff placement included paracetamol, ibuprofen and intranasal fentanyl, administered at the discretion of the emergency department (ED) clinician based on the dosing recommendation provided by the British National Formulary for Children.^18^ Following discharge from the ED, these children were followed up in the fracture clinic to monitor for displacement, complications and fracture union. Plain AP and lateral elbow radiographs were taken at presentation, after immobilisation, and at 1 and 3 weeks postinjury. Additional radiographs and follow-ups may be arranged by the treating clinician if deemed necessary. Reasons for placing a child into an above-elbow backslab instead of a collar and cuff included inadequate pain control and parental concerns to manage without a rigid cast.

**Figure 2:**
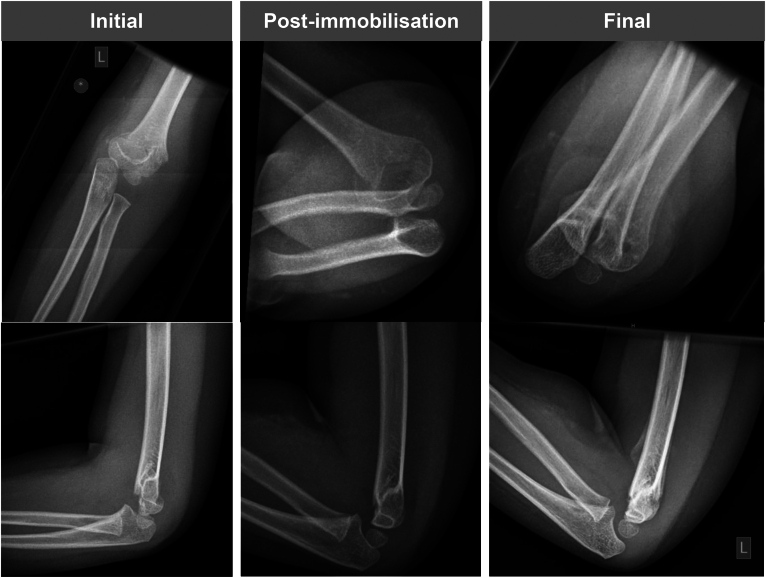
Initial, post-immobilisation and final radiographs

Fracture reduction was considered satisfactory only if the medial and lateral columns of the distal humerus were aligned on AP radiographs, and the AHL intersects the capitellum on lateral radiographs (Figure 2). Closed or open reduction and Kirschner wire fixation were offered if the fracture was not adequately reduced, or subsequently displaced with nonoperative treatment. The technique and construct of fixation was left to the discretion of the operating surgeon.

### Statistical analysis

Data were analysed using Statistical Package for the Social Sciences (SPSS) software version 28.0 (IBM, Armonk, NY, USA). Normality of distribution was assessed using the Shapiro–Wilk test. The paired t-test was used to compare the initial and post-immobilisation radiologic parameters with the final radiographs in the same patients. A *p*-value of <0.05 was deemed statistically significant.

## Results

**Figure 3:**
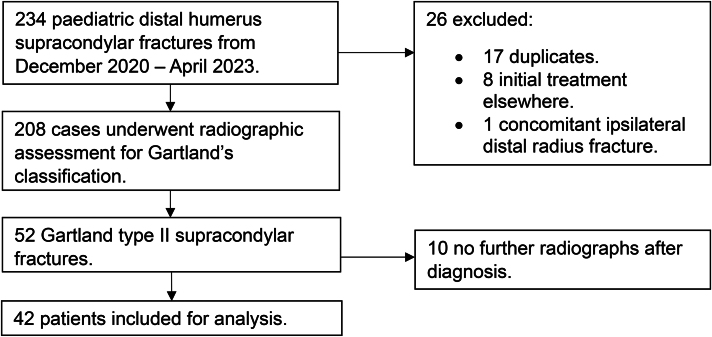
Patient inclusion flowchart

Figure 3 summarises the patient inclusion flowchart. None of the patients with a Gartland type II supracondylar fracture had an open injury or neurovascular compromise at presentation. Forty-two patients were included in the final analysis.

Table 1 summarises the demographic details of the included patients. The mean (sd) duration of radiographic follow-up was 5 (9) weeks. Patients were followed up in clinic for an average of 6 (9) weeks. The mean duration between data collection and date of injury was 17 (6) months.

**Table 1:** Patient demographics

Characteristics	*N* = 42
Sex, *n*	
Female	25
Male	17
Age in years, mean (SD)	6 (2)
Laterality, *n*	
Left	29
Right	13
Mechanism of injury, *n*	
High energy	10
Low energy	32

**Figure 4:**
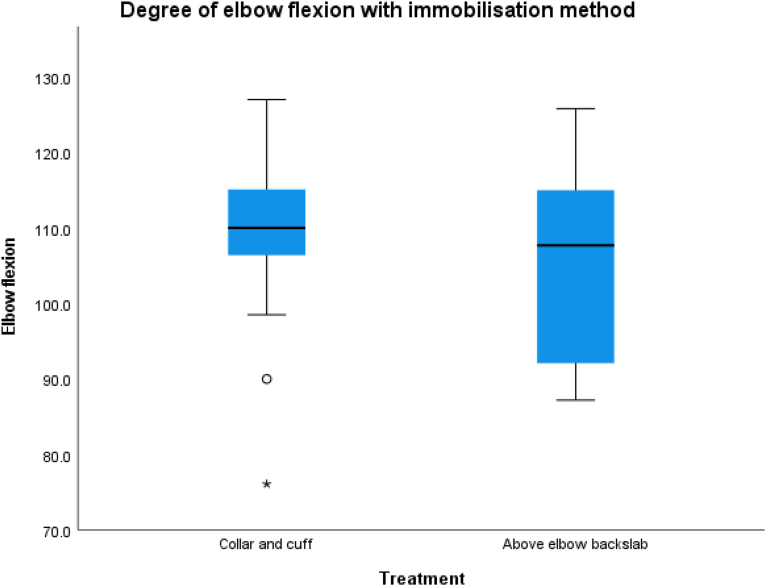
Degree of elbow flexion according to method of immobilisation

Thirty-four children were managed in a collar and cuff and six in an above-elbow backslab. The mean elbow flexion was 109.4 (9.2)° with a collar and cuff and 105.9 (14.6)° with an above-elbow backslab (Figure 4). Two patients underwent closed reduction and Kirschner wire fixation following unsatisfactory alignment in a collar and cuff.

There were no recorded complications in any children from their date of injury to the time of data collection. In particular, there were no cases of compartment syndrome or neurovascular compromise. No patients required a reoperation or corrective osteotomy.

Table 2 summarises the radiologic parameters on the initial, post-immobilisation and final radiographs. Application of a collar and cuff resulted in fracture reduction with improved Baumann's angle and LHCA at final radiographs (*p* = 0.035 and *p* < 0.001, respectively). However, only the difference in the LHCA reached the MCID. There was no significant change in the LHCA from immobilisation in a collar and cuff until final radiographic follow-up (*p*=0.274). Treatment in a collar and cuff was effective for patients aged <5 years and ≥5 years (Table 3), with significantly improved LHCA (*p* = 0.003 for age <5 years; *p* < 0.001 for age ≥5 years) and maintenance of LHCA from immobilisation until final radiographic follow-up (*p* = 0.662 for age <5 years; *p* = 0.143 for age ≥5 years). The differences between the initial and final Baumann's angle and LHCA were not statistically significant following immobilisation in an above-elbow backslab (*p* = 0.795 and *p* = 0.250, respectively).

**Table 2:** Radiologic parameters, mean (sd)

	Baumann's angle (°)			Lateral humeral–capitellar angle (°)		
	Initial	Post immobilisation	Final	Initial	Post immobilisation	Final
Collar and cuff	74.3 (10.1)	73.6 (6.9)	70.4 (7.0)	80.9 (13.1)	68.3 (11.3)	65.6 (11.2)
Above-elbow backslab	76.1 (12.5)	70.9 (2.2)	77.3 (3.1)	79.2 (19.0)	72.3 (21.8)	67.1 (19.0)
Overall	74.7 (10.1)	73.9 (6.6)	71.7 (6.9)	81.9 (14.6)	69.3 (13.1)	66.3 (12.3)

**Table 3:** Lateral humeral–capitellar angle with collar and cuff immobilisation based on age

Age (years)	Lateral humeral–capitellar angle (°), mean (sd)		
	Initial	Post collar and cuff immobilisation	Final
<5 (*n* = 13)	78.0 (14.1)	68.2 (11.2)	68.0 (12.3)
≥5 (*n* = 21)	82.7 (12.4)	68.3 (11.7)	64.5 (10.7)

## Discussion

Only two (4.8%) patients required closed reduction and Kirschner wire fixation in this study because of unsatisfactory alignment with nonoperative treatment. Thirty-four (81.0%) patients were managed in a collar and cuff with high elbow flexion without any recorded complications at a mean data follow-up of 17 months. In particular, there were no cases of compartment syndrome or neurovascular compromise. Furthermore, immobilisation in high flexion with a collar and cuff achieved a clinically and statistically significant improvement in the LHCA. There was no significant change in the LHCA between immobilisation and final radiographic follow-up.

To the best of our knowledge, this is the first study to report radiologic outcomes in children with Gartland type II supracondylar fractures managed in a collar and cuff with high elbow flexion without a formal closed reduction. Most studies on the nonoperative management of this injury included patients who underwent closed reduction, under conscious sedation or general anaesthetic, followed by immobilisation in a cast or collar and cuff.^9,10,13,16,19–21^ In our study, patients who were treated using a collar and cuff were placed into a mean elbow flexion of 109.4 (9.2)° without a formal reduction manoeuvre. In the majority of cases, this resulted in an acceptable reduction and maintained alignment until final radiographic follow-up, suggesting that this was an effective method of achieving and maintaining reduction.

In 2004, O’Hara *et al* proposed that all type IIB and III fractures require reduction and stabilisation with Kirschner wires because of the 29% risk of redisplacement.^6^ In their study, all 29 patients with a type IIA fracture achieved excellent outcomes with a full range of movement and a normal carrying angle.^6^ Among these 29 patients, 22 were treated with cuff or plaster immobilisation in flexion without closed reduction, whereas 7 underwent closed reduction because of an unacceptable humeral–capitellar angle.^6^ The authors did not encounter significant issues with soft tissue swelling with type IIA fractures and reported no incidence of vascular compromise or compartment syndrome, despite the theoretical risks associated with elbow flexion.^6,22^ Similarly, none of the patients in our study developed neurovascular compromise or compartment syndrome. We prefer to immobilise these patients in a collar and cuff, instead of an above-elbow cast, to avoid circumferential bandaging, accommodate soft tissue swelling and encourage an improvement in the LHCA. The low rate of complications indicates this is a safe immobilisation method for type II fractures. Caution should still be exercised for cases with features suggestive of a more severe soft tissue insult, such as severe swelling, ecchymosis or the pucker sign.^1^

Six patients in our study were managed in an above-elbow backslab. When compared against collar and cuff, immobilisation of undisplaced supracondylar fractures in an above-elbow backslab is associated with a shorter duration of pain and time taken to resume normal activity.^23^ The majority of patients in our study were compliant with 3 weeks of immobilisation in a collar and cuff, indicating that this was an acceptable treatment in most cases. Furthermore, treatment with a collar and cuff in flexion resulted in a significant improvement in the final LHCA. This did not reach statistical significance in those immobilised in an above-elbow backslab; however, this may be limited by the small number of patients in this group.

Only two (4.8%) patients in this study underwent closed reduction and Kirschner wire fixation. Both cases were because of unsatisfactory alignment on post-immobilisation radiographs taken in the ED. None of the patients in whom satisfactory alignment was achieved lost position in a collar and cuff. Pandey *et al* reported a 10% rate of patients requiring open reduction and Kirschner wire fixation following attempted nonoperative treatment with closed reduction and above-elbow backslab.^20^ Pierantoni *et al* reported a 16.1% displacement rate following closed reduction and above-elbow cast immobilisation requiring secondary closed reduction and Kirschner wire fixation.^21^ Parikh *et al* reported redisplacement in 7 of 25 (28%) patients managed with closed reduction and cast immobilisation, 5 of whom underwent subsequent closed reduction and Kirschner wire fixation.^13^ In our series, none of the patients developed significant loss of reduction following satisfactory post-immobilisation radiographs taken in the ED. There were no significant changes in the Baumann's angle and LHCA post immobilisation in a collar and cuff until final radiographic follow-up, supporting this treatment modality as an effective method for immobilisation. Nonetheless, as per the recommendations made by Parikh *et al*, we closely monitored these patients with interval radiographs taken at 1 and 3 weeks, with a view to selectively fix those that lose reduction.^13^

Moraleda *et al* evaluated the clinical outcomes of 46 type II supracondylar fractures treated with a long arm splint without reduction.^11^ They reported a mild decrease in elbow flexion and a mild elbow hyperextension, although functional results were satisfactory in the majority of patients.^11^ However, 12 patients had a cubitus varus deformity, 3 of whom underwent corrective osteotomy.^11^ By contrast, there were no cases that required corrective osteotomy in our study cohort. We immobilised patients with a mean elbow flexion of >100°, whereas Moraleda *et al* treated patients in a splint at <90°.^11^ It has been suggested that 120° of elbow flexion is required to maintain stable reduction.^24^ Although our patients were also treated without a formal attempt at reduction, our results suggest that immobilisation with a higher degree of elbow flexion may improve alignment and reduce the incidence of clinically significant cubitus varus requiring surgical intervention.

### Study limitations

A limitation of this study was the lack of functional outcomes scores in these patients. In addition, we did not measure and compare the carrying angles of the injured elbow with the contralateral limb to assess for cubitus varus or valgus deformity. This information would have been useful to evaluate the long-term impact of this treatment strategy and represents an area for future research. Nonetheless, we suspect that most patients achieved a satisfactory function, as otherwise we would have expected a higher rate of re-presentation to orthopaedics. The lack of any adverse events recorded at the time of data collection, which was at an average of 17 months post injury, indicates that most of our patients were unlikely to have experienced any significant acute or chronic complications from their injuries. However, a larger sample size may be required to adequately assess the rate of complications, such as neurovascular compromise, compartment syndrome and need for subsequent surgery.

## Conclusion

Immobilisation with a collar and cuff in high elbow flexion is a safe and effective nonoperative treatment option for reducing and immobilising Gartland type II supracondylar fractures. This could be easily applied in the ED without a formal closed reduction. Surgical treatment could be reserved for cases with an unsatisfactory alignment or early loss of reduction following attempted nonoperative treatment.
